# Supplementary material for: Response of an actin network in vesicles under electric pulses
Source: Sci Rep. 2019 May 31;9:8151. doi: 10.1038/s41598-019-44613-5 (PMC6544639; doi:10.1038/s41598-019-44613-5)
Supplement: Supplementary file 1 — Supplementary Material [file 41598_2019_44613_MOESM1_ESM.pdf]

Supplementary material for "Response of an actin network in vesicles under electric pulses"

Dayinta L. Perrier[1], Afshin Vahid[1], Vaishnavi Kathavi[1], Lotte Stam[1], Lea Rems[1], Yuval Mulla[*2], Aswin Muralidharan[1], Gijsje H. Koenderink[2], Michiel T. Kreutzer[1], and Pouyan E. Boukany[1]

[1]Department of Chemical Engineering, Delft University of Technology, the Netherlands
[2]AMOLF, Department of Living Matter, the Netherlands

[*]Current Address: Institute for Biological Physics, University of Cologne, Germany

S. 1 Movies

Supplementary movie S1. The empty GUV of Figure 2B during a 500 μs pulse of 220 V/mm. The scale bar in the movie is 5 μm. The actual duration of the movie is 19.62 ms.

Supplementary movie S2. The actin-encapsulated GUV of Figure 2C during a 500 μs pulse of 130 V/mm. The scale bar in the movie is 5 μm. The actual duration of the movie is 5.45 ms.

Supplementary movie S3. The disruption of the actin network of the actin-encapsulated GUV shown in Figure 4B. The GUV membrane is shown in red, and the actin network in green. The scale bar in the movie is 5 μm. The movie is sped up 34 times, the actual duration is 378 s.

S. 2 Visualization of the actin shell

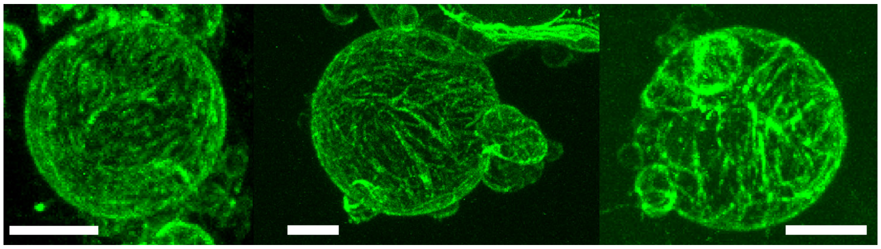

Figure S. 1: 3D brightest point projection of confocal images of the formed actin network. For these images, only the actin is fluorescently labeled. The length of filament bundles in the GUVs is between 1 and 5 μm and the thickness varies from 0.5 to 1.5 μm (see Fig. S5 for more details). The number of slices are 125, 178 and 141 for the left, middle and right images, respectively. The distance between the slices (z-step) is 100 nm for each image. The scale bar in all images is 5 μm.

S. 3 Actin polymerization outside of the GUV

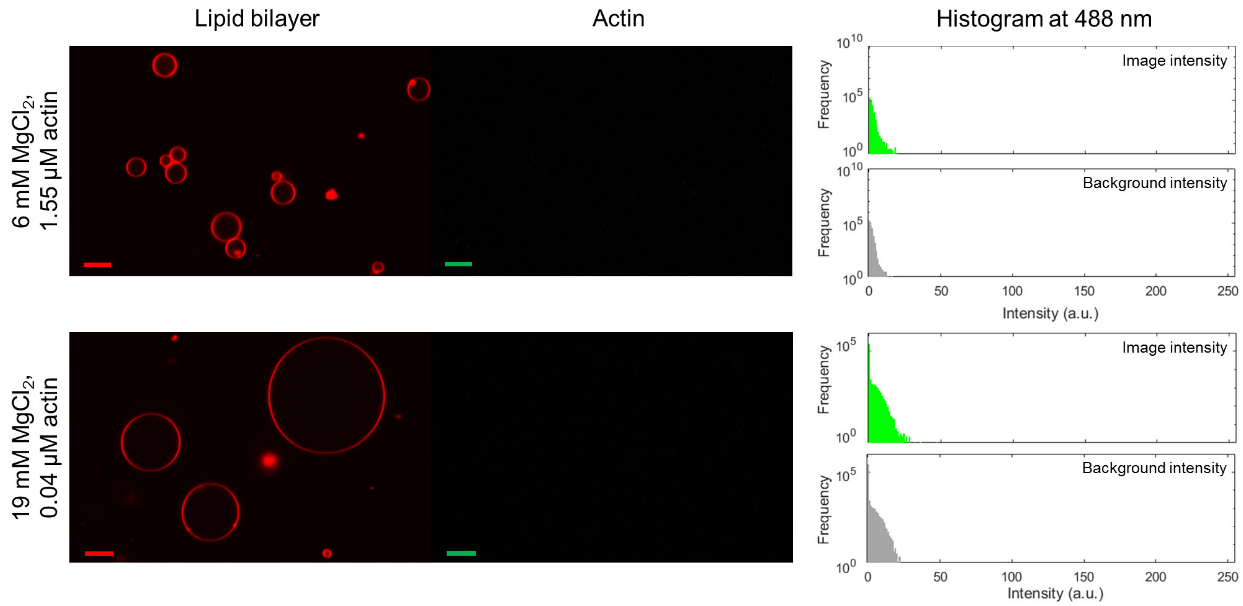

Figure S. 2: Actin polymerization on the outside of empty GUVs. *Top* : Empty GUVs with an outside concentration of actin and $MgCl_2$ of the overnight-conditions (1.55 μM actin and 6 mM $MgCl_2$). *Bottom* : Empty GUVs with an outside concentration of actin and $MgCl_2$ during the electroporation experiments (0.04 μM actin and 19 mM $MgCl_2$). The red fluorescence signal shows the membrane, and the green fluorescence signal shows the actin. The graph on the right displays the histograms of the intensity at 488 nm, the excitation wavelength for the actin network. The background intensity is obtained from empty GUVs in the absence of actin, imaged at the same conditions. In both cases, no polymerization of the actin monomers on the outside of the GUVs and no significant increase in the intensity at 488 nm is observed. The scale bar in all images is 10 μm.

S. 4 Photobleaching correction

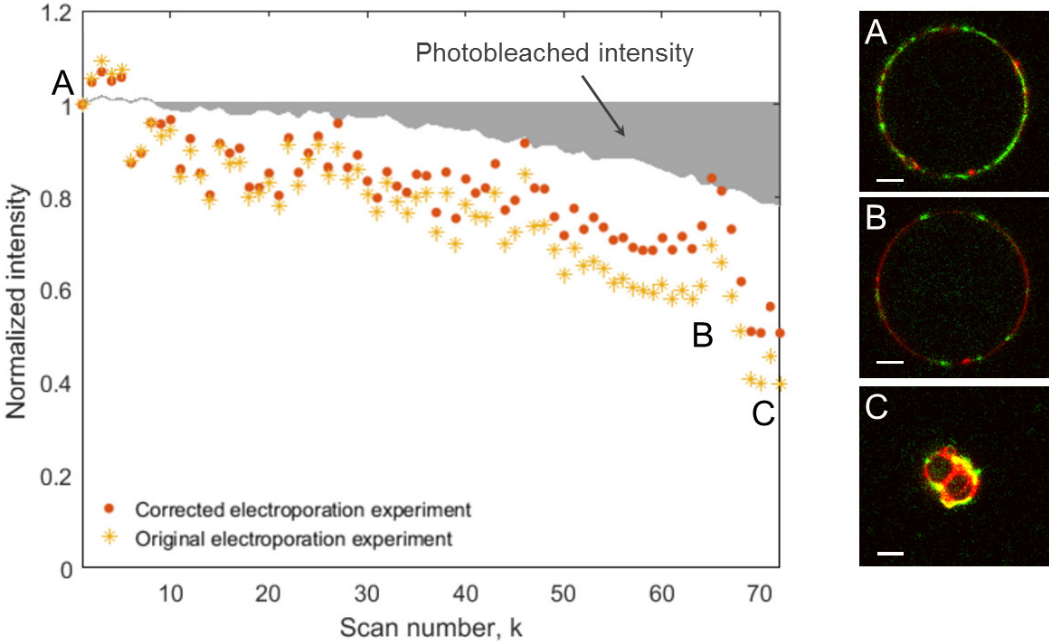

Figure S. 3: (Left) The corrected intensity loss of the actin, normalized by the initial intensity, during a typical electroporation experiment for photobleaching, as discussed in the main text. The time interval between the scans is 7.07 seconds. (Right) Three images at different times during the experiment, indicated in the graph (A, B and C), where the signal of the membrane is depicted in red and the signal of the actin network in green. The scale bar in all images is 5 μm.

S. 5 Background signal of confocal experiments

The fluorescent signal of the actin network was obtained by determining the gray value of a square surface that fits the maximum size of the GUV in the different imaging planes (indicated in red in Figure S. 4). Therefore, the intensity decrease that we observed upon the application of electric pulses includes both the actin network and the actin present in the bulk of the GUVs. Based on our estimates of the relevant forces acting on the filaments during the pulse, electrophoretic forces can disrupt the actin network. Consequently, one would expect that either the intensity in the bulk of the GUV increases (when the actin remains in the GUV), or the intensity on the outside of the GUV increases if the membrane is permeabilized (i.e. the background). However, since the GUV concentration was dilute and the expelled actin could diffuse freely, we did not detect any increase in the fluorescence of the background. The fluorescence intensity of the bulk actin inside the GUV can only be determined on the equatorial plane (indicated in blue in Figure S. 4), whereas the fluorescent intensity of the actin network can be obtained from the complete z-stack. Consequently, the mean intensity of the GUV is dominated by the fluorescent signal of the actin network, as can be seen in Figure S. 4B. As the electroporation experiments continued, because of the photobleaching, the total actin signal was decreased, as can be seen in Figure S. 4C. Therefore, both the background fluorescent intensity and the inside fluorescent intensity approach the noise level of our camera. We were therefore unable to conclude whether the actin remains in the bulk of the GUV or is expelled. We did not observe any re-polymerization in any experiments at higher electric fields (> 150 V/mm), suggesting that some actin might have been expelled which lowers the concentration on the inside of the GUV below the critical concentration for polymerization. However, it must be noted that as the GUVs were permeabilized by the electric pulse, other side effects might affect the re-polymerization of actin network (such as local changes in pH or Mg^{2+} concentration).

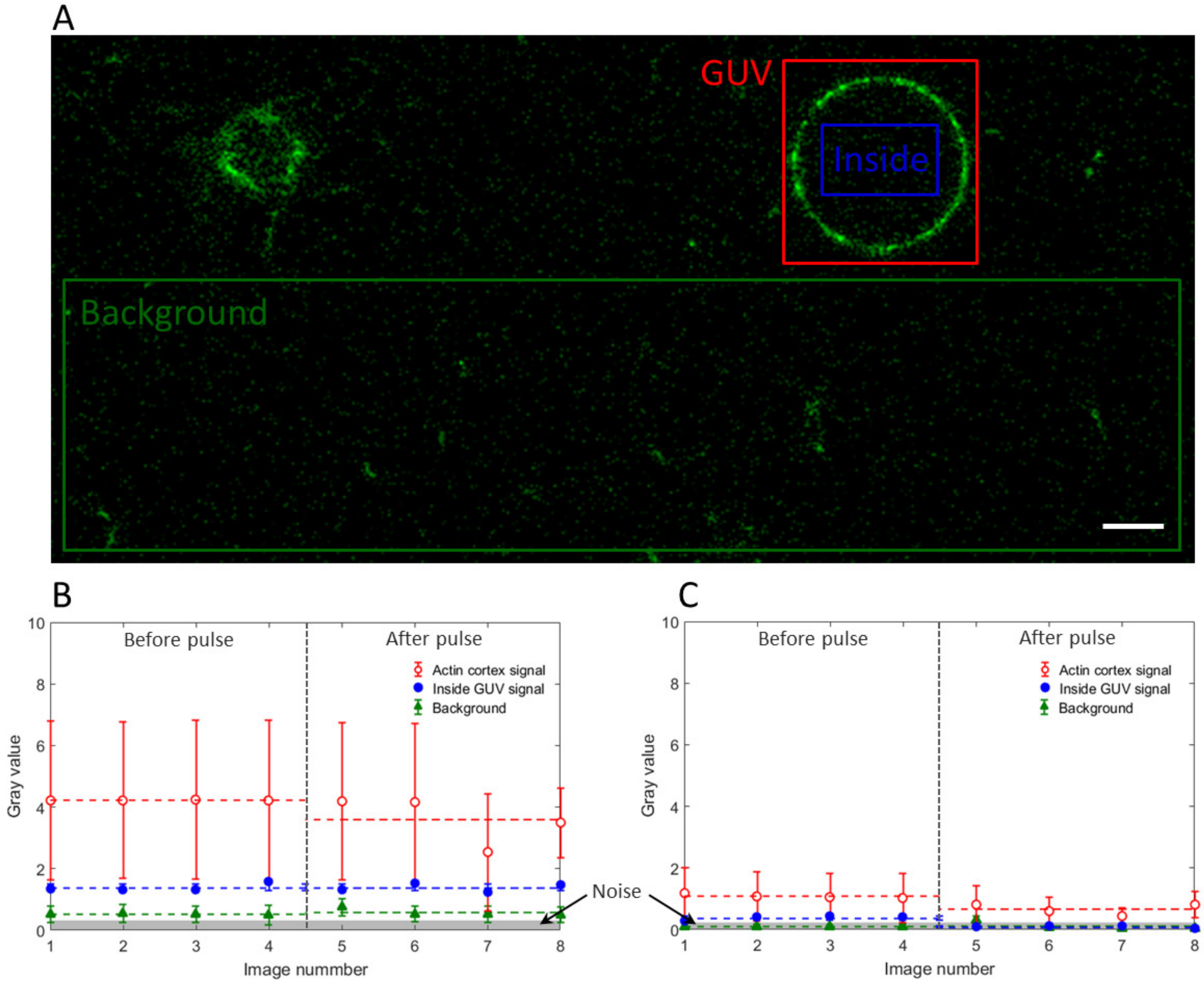

Figure S. 4: The gray levels of different regions of the confocal images of the middle plane. (A) A confocal image showing the areas of interest: background (green), inside of the GUV (blue) and the square area around the GUV (red). (B) The gray values for the three regions of interest during the first pulse ($\sim$ 100 V/mm). (C) The gray values for the three regions of interest during the last pulse ($\sim$ 320 V/mm). The dotted lines indicate the mean values before and after the pulse. The scale bar is 5 μm.

S. 6 Thickness of the actin network

Figure S. 5A shows that the actin shell thickness was not constant and varied per GUV. The thickness of the shell inside the GUVs was estimated based on the intensity profile measurements of a fluorescent shell by the full width at half-maximum (FWHM) method. The actin shell thickness was measured at four different pole locations per GUV. First, the fluorescence intensity profile of the actin shell (Alexa Fluor 488 actin signal: ex/em 495 nm/519 nm) was extracted across the actin shell based on confocal microscopy measurements (see Figure S. 5B). Then, the intensity profile was fitted to a FWHM of Gaussian function:

$$y = y_0 + \frac{A \exp\left[\frac{-4\log(2)(x-x_c)^2}{w^2}\right]}{w\sqrt{\frac{\pi}{4\log(2)}}}, \tag{S. 1}$$

where w is the full width at half maximum, y_0 is the background intensity, x_c is the center of the Gaussian fit (the position of peak), and A is the profile area under the curve of the Gaussian fit. The inset shows a confocal fluorescence image at the mid-plane plane of the GUV (highlighted area), where the intensity profile was obtained. This measurement was repeated for four poles (top, bottom, left, and right) per GUV. The discontinuity in the actin signal is created by that in the actin shell, which could be caused by the location of the ionophore inside the membrane.

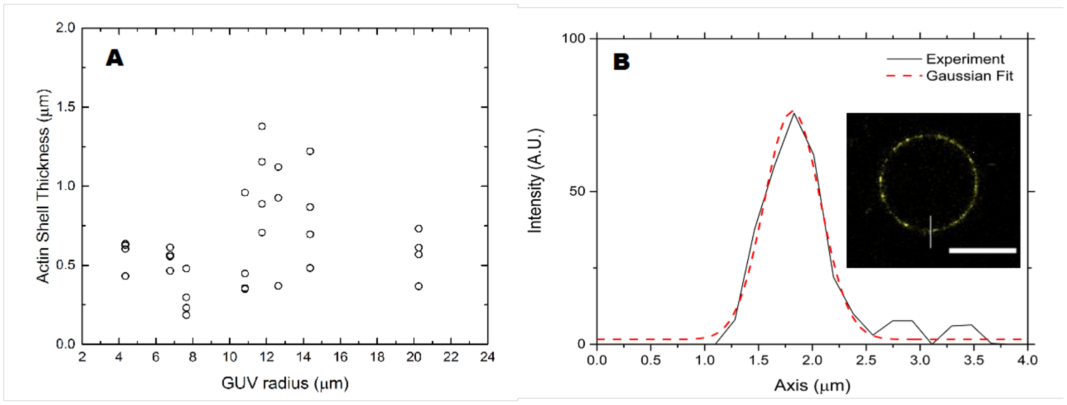

Figure S. 5: (A) Actin shell thickness as a function of GUV size. The shell thickness was measured based on the intensity profile measurements of a fluorescent membrane shell at four different pole locations of the GUV by using the full width at half-maximum (FWHM) method. (B) Fluorescence intensity profile of the actin shell (solid line) was obtained along the white line (thin vertical line in the inset). The dashed line shows a Gaussian fit. The inset shows a confocal fluorescence image at the mid-plane of the GUV. The scale bar is 10 μm.

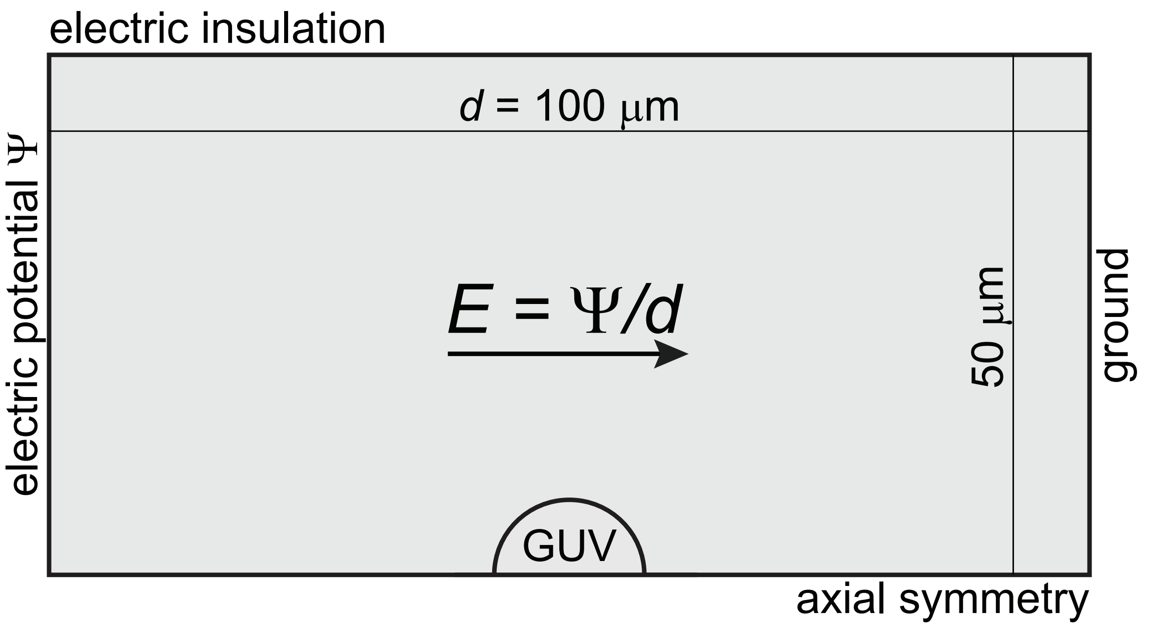

Figure S. 6: A 2-D schematic geometry of a spherical GUV exposed to an electric field **E** inside a cylindrical domain representing the external liquid.

S. 7 Electric field inside an electroporated GUV

We numerically calculated the electric field inside an electroporated GUV using Comsol Multiphysics software, similarly as in previous works (1, 2). The model of a GUV exposed to an electric field E is built in a 2D axisymmetric coordinate system (Figure S. 6). A spherical GUV is positioned inside a cylindrical domain representing the external liquid. The exposure of the GUV to an electric pulse is modeled by assigning an electric potential to two opposite sides of the external domain (Figure S. 6). The electric potential distribution Ψ is calculated by

$$\nabla . \left[\left(\lambda_{\mathrm{i,e}} + \epsilon_{\mathrm{i,e}} \frac{\partial}{\partial \mathrm{t}} \right) \nabla \Psi \right] = 0, \tag{S. 2}$$

where $\lambda_{\mathrm{i,e}}$ and $\epsilon_{\mathrm{i,e}}$ denote, respectively, the conductivity and the dielectric permittivity of the internal (subscript i) or external (subscript e) liquid. The GUV membrane is modelled via a boundary condition, which describes the continuity of the normal component of the electric current density **J** across the membrane

$$\mathbf{n}.\mathbf{J} = \left[\mathrm{G_m} + \mathrm{C_m} \frac{\partial}{\partial \mathrm{t}} \right] \Psi_{\mathrm{m}} \tag{S. 3}$$

where **n** denotes the unit vector normal to the membrane surface, and $\mathrm{G_m}$ and $\mathrm{C_m}$ denote the membrane conductance and capacitance, respectively. The transmembrane voltage

Ψ_m corresponds to the difference between the electric potentials on the two sides of the membrane. Membrane electroporation is included into the model by solving the ordinary differential equation, which describes the density of electropores created in the membrane under the influence of the induced transmembrane voltage (3)

$$\frac{\mathrm{dN}}{\mathrm{dt}} = \alpha \exp\left(\frac{\Psi_m^2}{V_{ep}^2}\right)\left(1 - \frac{N}{N_0}\exp\left(-q\frac{\Psi_m^2}{V_{ep}^2}\right)\right) \tag{S. 4}$$

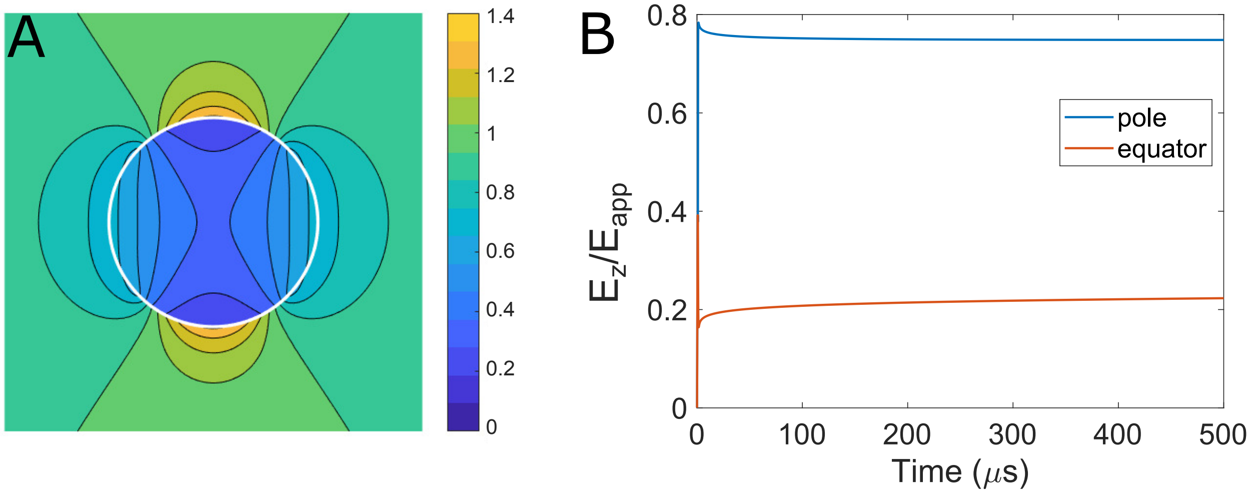

Figure S. 7: Magnitude of the electric field induced inside and around a GUV of size 10 μm exposed to an electric field. The magnitude of the induced electric field is normalized to the magnitude of the applied electric field of 150 V/mm. The applied electric field is directed from left to right. As shown, the highest field is induced around the poles.

Since electropores conduct ions, they increase the membrane conductance, which in turn affects the transmembrane voltage. The increase in membrane conductance due to electropores is(2)

$$\mathrm{G_{ep}} = \mathrm{N}\frac{2\pi \mathrm{r_p^2}\lambda_\mathrm{p}}{\pi \mathrm{r_p} + 2\mathrm{d_m}} \tag{S. 5}$$

where r_p, λ_p, and d_m are the pore radius, conductivity of the solution inside the pore, and membrane thickness, respectively. The total membrane conductance G_m in equation (S. 3) is calculated at each time step as the sum of the passive membrane conductance G_{m0} and the conductance due to electropores G_{ep}. The values of the model parameters are given in Table S. 1. Figure S. 7A shows the magnitude of the electric field inside and around the GUV at the end of 150 V/mm pulse (500 μs). Note that the electric field values are normalized with respect to the applied electric field, $E_{app} = 150$ V/mm. Figure S. 7B depicts the electric field inside the GUV at two points beneath the membrane. The first point is located at the pole of the GUV (where the membrane normal is parallel to the applied electric field) and the second point is located at the equator of the GUV (where the membrane normal is perpendicular

Table S. 1: Model parameters.

**Parameter**	**Symbol**	**Value**	**Reference**
GUV radius	R	10 μm	experiment
Electric conductivity of internal and external fluid	$\lambda_i\ \lambda_e$	0.3 S/m	*
Dielectric permittivity of internal and external fluid	ϵ_i, ϵ_e	80	Chan et al. (4)
Passive membrane conductance	$G_{m0} = \lambda_{m0}/d_m$	0.25 S/m^2	Chan et al. (4)
Membrane conductivity	λ_{m0}	10^{-9} S/m	Chan et al. (4)
Membrane capacitance	C_m	0.7 μF/cm^2	Chan et al. (4)
Membrane thickness	d_m	4 nm	Chan et al. (4)
Conductivity of solution inside the pore	$\lambda_p = \lambda_i = \lambda_e$	0.3 S/m	*
Pore radius	r_p	1 nm	Debruin (3)
Electroporation parameter	α	10^9 m^{-2}s^{-1}	Debruin (3)
Characteristic voltage of electroporation	V_{ep}	0.258 V	Debruin (3)
Electroporation constant	q	2.46	Debruin (3)
Equilibrium pore density	N_0	15×10^9 m^{-2}	Debruin (3)
Applied electric field	E_{app}	150 V/mm	experiment
Pulse duration	t_{pulse}	500 μs	experiment
Pulse rise time	t_{rise}	1 μs	arbitrary

*The conductivity was estimated as the conductivity of an aqueous solution of 19 mM $MgCl_2$; we took tabulated values (5) for 53 mM (0.5% mass) $MgCl_2$ and 106 mM (1.0% mass) $MgCl_2$ and used linear extrapolation to obtain the conductivity of 19 mM $MgCl_2$.

to the applied electric field). At both points the electric field is directed parallel to the applied electric field. As shown, the maximum electric field induced inside the GUV after the electroporation is about $0.2\,E_{app}$ and $0.8\,E_{app}$ for the vesicle size of $R = 10\,\mu m$.

S. 8 Actin intensity versus transmembrane voltage

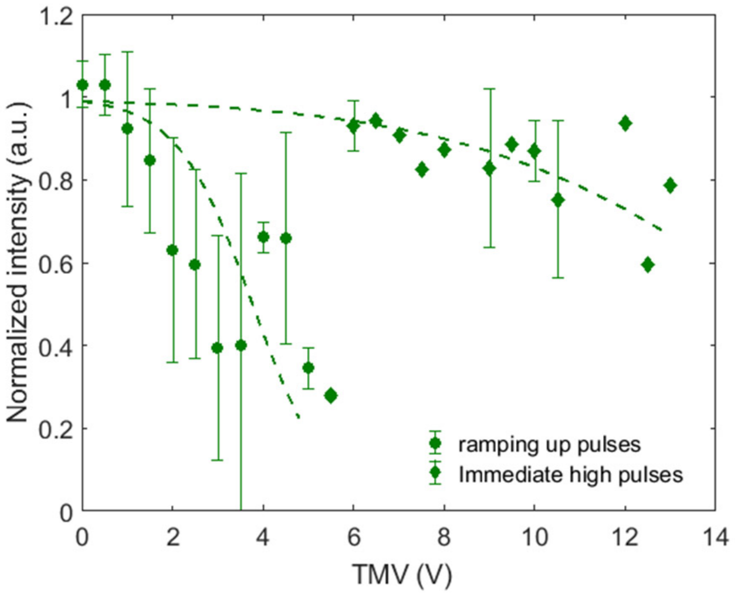

Figure S. 8: The averaged normalized intensity of the actin network of all GUVs plotted against the calculated transmembrane voltage (TMV). Since the transmembrane voltage cannot increase further as soon as the membrane is permeabilized, the estimated TMV is a theoretical value which is obtained using $\Psi_{\mathrm{m}} = 1.5\,\mathrm{R}\,\mathrm{E}$, given in the main text. The GUVs have been exposed to ramping up pulses (ranging from $10 - 300$ V/mm with steps of approximately 10 V/mm) and immediate high pulses (ranging from $300 - 1000$ V/mm with steps of approximately 200 V/mm), where only 2 to 4 pulses were applied of increasing field. The graph shows that the intensity decreases above the critical TMV of 1 V, which is in the electroporative regime.

S. 9 Joule heating during electroporation of GUVs

We observed that the decrease in the fluorescence signal of the actin is associated with a breakdown of the actin shell inside of GUVs (between E=150 V/mm and E=300 V/mm, see Figure 5B). To make sure that actin-encapsulated GUVs were not disrupted due to Joule heating from the electric pulse, we estimated the maximum amount of Joule heating in our experiments at E = 300 V/mm. We calculated the maximum temperature rise during an electric pulse with $t_{pulse} = 500\mu s$:

$$\Delta T = \frac{E^2 \lambda_e}{c_m \rho} t_{pulse} = \frac{(3.0 \times 10^5 \frac{V}{m})^2 \times 0.3 \frac{S}{m}}{4200 \frac{J}{kgK} \times 1000 \frac{kg}{m^3}} \times 5.10^{-4} s \sim 3K$$

where ΔT is the temperature rise (in K), c_m is the heat capacity of the sample, E is the electric field intensity, t_{pulse} is the pulse duration, λ_e is the electrical conductivity of sample, and ρ is the mass density of the sample. The heat capacity and density of the sample are taken to be the the same as for water. The electrical conductivity of the solution is taken from Table S1. The calculated temperature is still small (in the range of imposed electric fields between 150 V/mm and 300 V/mm) and we can therefore reasonably expect that it did not influence the actin-encapsulated GUVs. However, the effect of Joule heating will be present for the higher applied electric fields (E=800 V/mm) and cannot be neglected.

S. 10 P-value analysis for the response of the empty and actin-encapsulated GUVs

As shown in Figure S. 9, we have estimated P-values between immediate and ramping-up experiments in Figure 5 in the main text for both empty and actin-encapsulated GUVs. When E=200-260 V/mm, the P-value is significantly larger than 0.1 (P=0.21), so they are not significantly different. However at E=280-380 V/mm, the P-value is 0.07 , and the difference can be significant (P<0.1). In case of actin-encapsulated GUVs, the difference between immediate and ramping-up pulses is significant for both loss of area and loss of intensity at E=290-400 V/mm (P<0.05).

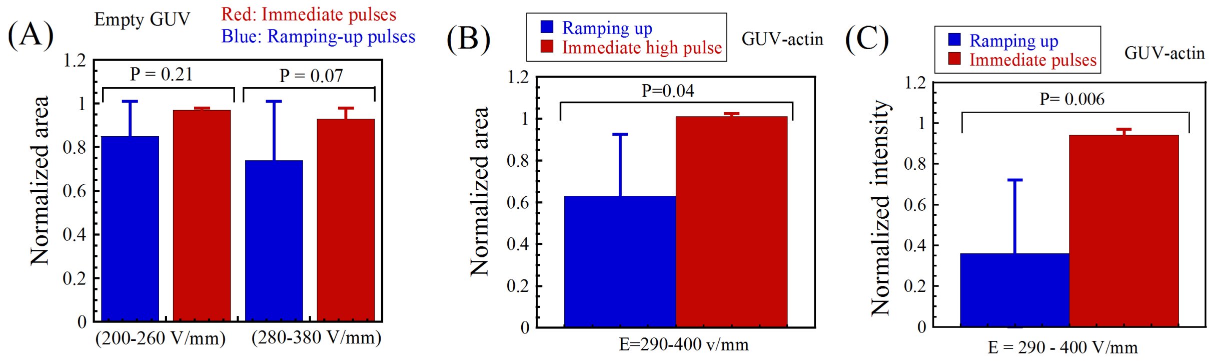

Figure S. 9: Effect of pulsing condition (immediate pulse versus ramping-up) on (A) the loss of area in empty GUVs at E =200-260 V/mm and E =280-380 V/mm, and (B-C) the loss of area and intensity in GUVs (encapsulated with actin) at E =290-400 V/mm.

References

[1] Retelj, L., G. Pucihar, and D. Miklavčič. 2013. Electroporation of intracellular liposomes using nanosecond electric pulses–a theoretical study. *IEEE Transactions on Biomedical Engineering.* 60:2624–2635.

[2] Rems, L., M. Ušaj, M. Kandušer, M. Reberšek, D. Miklavčič, and G. Pucihar. 2013. Cell electrofusion using nanosecond electric pulses. *Scientific reports.* 3:3382.

[3] DeBruin, K. A., and W. Krassowska. 1999. Modeling electroporation in a single cell. i. effects of field strength and rest potential. *Biophysical journal.* 77:1213–1224.

[4] Chan, K. L., P. R. Gascoyne, F. F. Becker, and R. Pethig. 1997. Electrorotation of liposomes: verification of dielectric multi-shell model for cells. *Biochimica et Biophysica Acta (BBA)-Lipids and Lipid Metabolism.* 1349:182–196.

[5] Haynes, W. M. 2014. CRC handbook of chemistry and physics. CRC press.
